# Performance of the 2023 Duke-International Society of Cardiovascular Infectious Diseases Diagnostic Criteria for Infective Endocarditis in Relation to the Modified Duke Criteria and to Clinical Management—Reanalysis of Retrospective Bacteremia Cohorts

**DOI:** 10.1093/cid/ciae040

**Published:** 2024-02-08

**Authors:** Helena Lindberg, Andreas Berge, Martin Jovanovic-Stjernqvist, Malin Hagstrand Aldman, David Krus, Jonas Öberg, Fredrik Kahn, Anna Bläckberg, Torgny Sunnerhagen, Magnus Rasmussen

**Affiliations:** Department of Infectious Diseases, Hospital of Halland, Halmstad, Sweden; Division of Infection Medicine, Department of Clinical Sciences Lund, Lund University, Lund, Sweden; Unit of Infectious Diseases, Department of Medicine, Karolinska Institutet, Stockholm, Sweden; Department of Infectious Diseases, Karolinska University Hospital Stockholm, Sweden; Division of Infection Medicine, Department of Clinical Sciences Lund, Lund University, Lund, Sweden; Division of Infection Medicine, Department of Clinical Sciences Lund, Lund University, Lund, Sweden; Department of Infectious Diseases, Skåne University Hospital Lund, Sweden; Division of Infection Medicine, Department of Clinical Sciences Lund, Lund University, Lund, Sweden; Department of Infectious Diseases, Skåne University Hospital Lund, Sweden; Division of Infection Medicine, Department of Clinical Sciences Lund, Lund University, Lund, Sweden; Department of Infectious Diseases, Helsingborg Hospital, Helsingborg, Sweden; Division of Infection Medicine, Department of Clinical Sciences Lund, Lund University, Lund, Sweden; Department of Infectious Diseases, Skåne University Hospital Lund, Sweden; Division of Infection Medicine, Department of Clinical Sciences Lund, Lund University, Lund, Sweden; Department of Infectious Diseases, Skåne University Hospital Lund, Sweden; Division of Infection Medicine, Department of Clinical Sciences Lund, Lund University, Lund, Sweden; Clinical Microbiology and Infection Control, Region Skåne Office for Medical Services, Lund, Sweden; Division of Infection Medicine, Department of Clinical Sciences Lund, Lund University, Lund, Sweden; Department of Infectious Diseases, Skåne University Hospital Lund, Sweden

**Keywords:** infective endocarditis, diagnostic criteria, validation, bacteremia

## Abstract

**Background:**

Revised diagnostic criteria for infective endocarditis (IE), the 2023 Duke-ISCVID criteria, were recently presented and need validation. Here, we compare the 2000 modified Duke criteria for IE with Duke-ISCVID among patients with bacteremia and relate the diagnostic classification to IE treatment.

**Methods:**

We reanalyzed patient cohorts with *Staphylococcus aureus*, *Staphylococcus lugdunensis*, non–β-hemolytic streptococci, *Streptococcus*-like bacteria, *Streptococcus dysgalactiae, Enterococcus faecalis,* and HACEK (*Haemophilus*, *Aggregatibacter*, *Cardiobacterium*, *Eikenella*, *Kingella*) bacteremia. Episodes were classified as definite, possible, or rejected IE with the modified Duke and Duke-ISCVID criteria. Reclassification included the microbiology criteria, positron emission tomography–computed tomography, and cardiac implanted electronic devices. To calculate sensitivity, patients treated for IE were considered as having IE.

**Results:**

In 4050 episodes of bacteremia, the modified Duke criteria assigned 307 episodes (7.6%) as definite IE, 1190 (29%) as possible IE, and 2553 (63%) as rejected IE. Using the Duke-ISCVID criteria, 13 episodes (0.3%) were reclassified from possible to definite IE, and 475 episodes (12%) were reclassified from rejected to possible IE. With the modified Duke criteria, 79 episodes that were treated as IE were classified as possible IE, and 11 of these episodes were reclassified to definite IE with Duke-ISCVID. Applying the decision to treat for IE as a reference standard, the sensitivity of the Duke-ISCVID criteria was 80%. None of the 475 episodes reclassified to possible IE were treated as IE.

**Conclusions:**

The Duke-ISCVID criteria reclassified a small proportion of episodes to definite IE at the expense of more episodes of possible IE. Future criteria should minimize the possible IE group while keeping or improving sensitivity.

## Teaser

The new Duke-ISCVID criteria for infective endocarditis reclassified 12% of episodes in a cohort of patients with bacteremia (n = 4050) from rejected to possible endocarditis. Only 11 of 79 episodes of possible endocarditis, treated as endocarditis, were reclassified to definite endocarditis.

## Graphical Abstract

**Graphical Abstract:**
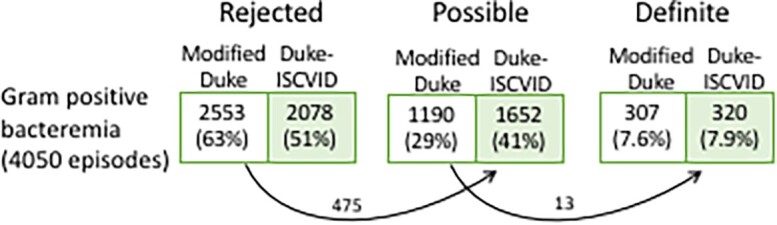

**(See the Invited Commentary by Chambers et al. on pages 964–7.)**

Infective endocarditis (IE) is a severe disease affecting the heart valves where the typical lesion, the vegetation is a hallmark for the condition. Whereas surgery or autopsy offers the possibility for an undisputable diagnosis of IE, the clinical diagnostic criteria for IE have varied over time. A landmark in the history of criteria development was when Durack and coworkers introduced echocardiography as an important major criterion in the Duke criteria in 1994 [1]. The Duke criteria were then modified in the year 2000, after validation in a cohort of 800 patients with IE, with an aim to lower the numbers of “possible IE,” and thus increase the specificity [2]. A combination of 1 major with 1 minor criterion or ≥3 minor criteria alone were introduced as the “floor” for possible IE. In the 2015 European Society of Cardiology criteria [3], further imaging modalities, including fluorine 18 fluorodeoxyglucose positron emission tomography (PET)–computed tomography (CT) and cardiac CT were added to define major imaging criteria.

The recently presented 2023 Duke-ISCVID (hereafter referred to as Duke-ISCVID) criteria have kept the original structure of the 1994 Duke criteria, with major and minor criteria [4]. The revisions were based on opinions from a group of experts and not on systematic literature review or analysis of new cohorts. Thus, validation studies and continuous updates are called for. The Duke-ISCVID criteria have added several bacteria to the list of typical IE pathogens [4]. In addition, the presence of a cardiovascular implanted electronic device (CIED) was added as a new predisposition minor criterion. Importantly, PET-CT and cardiac CT were introduced as imaging techniques for major imaging criteria, in concordance with the ESC criteria from 2015 [3], and several features of the criteria were explained more precisely. The main aim of the Duke-ISCVID criteria for IE was to provide a relevant update on imaging criteria and bacteriology [4].

One particular clinical situation in which IE needs to be considered is the finding of gram-positive or HACEK (*Haemophilus*, *Aggregatibacter*, *Cardiobacterium*, *Eikenella*, *Kingella*) bacteria in blood cultures. For patients with such bacteremia, the risk for IE needs to be evaluated so that echocardiographic investigations are performed in patients at risk for IE. The finding of IE has direct implications for prolonged treatment time and also for the decision about heart valve surgery. Therefore, several studies have been performed to determine risk factors for IE and risk stratification systems for IE in patients with bacteremia caused by gram-positive bacteria, such as *Staphylococcus aureus* [5–7], viridans streptococci (also known as non–β-hemolytic streptococci, NBHS) [8–10], and *Enterococcus faecalis* [11–13].

The objective of the current study was to compare the performance of the Duke-ISCVID criteria for IE [4] with that of the 2000 modified Duke criteria [2] in patients with bacteremia from 14 different cohorts. The study also evaluated the agreements of the classifications by the sets of criteria compared with the clinical decision to treat as IE.

## METHODS

Fourteen retrospective, population-based cohorts of patients with bacteremia have been published by our research group (Table 1). The cohorts were gathered from 3 geographically defined regions in Sweden and were population based. All studies included consecutive patients with bacteremia with a given bacterium or group of bacteria. The observation time for each episode was ≥1 year. In Table 1, the underlying population is given for the relevant time periods. Classification of IE status had been performed using the 2000 modified Duke criteria [2] or the 2015 European Society of Cardiology criteria [3] with some modifications with regard to the classification of *Streptococcus-*like bacteria [18]. For all cohorts, the absence of information about a particular feature was regarded as if that factor was missing. No imputations were made with regard to features included in the different IE criteria. A reevaluation of data from these cohort was performed, and each patient was classified as having rejected, possible, or definite IE according to the 2000 modified Duke criteria [2] and the Duke-ISCVID criteria, and the number of reclassifications was determined.

**Table 1.** Details of the Bacteremia Patient Cohorts

Bacteria	Period	Region in Sweden	Population (in Millions)	Reference	Bacteremia Episodes, No.	Episodes With IE, No. (%)
*Staphylococcus aureus*	2016	Skåne	1.32	[6]	542	40 (7.4)
	2017	Skåne	1.34	[6]	556	29 (5.2)
	2017–2019	Halland	0.32–0.33	[14]	267	24 (9.0)
*Staphylococcus lugdunensis*	2015–2019	Skåne	1.30–1.38	[15]	65	5 (7.7)
NBHS^a^	2012–2013^b^	Skåne	1.26–1.27	[10]	312	20 (6.4)
	2015–2016^c^	Skåne	1.30	[16]	244	27 (11.1)
NBHS^d^	2017–2019	Halland	0.32–0.33	[14]	154	10 (6.5)
*Streptococcus bovis* ^e^	2003–2018	Skåne	1.15–1.36	[17]	210	28 (13.3)
*Streptococcus*-like bacteria^f^	2012–2017	Skåne and Stockholm	3.2	[18]	568	32 (5.6)^g^
*Streptococcus dysgalactiae*	2015–2018	Skåne	1.30–1.36	[19]	287	4 (1.4)
*Enterococcus faecalis*	2017–2019	Halland	0.32–0.33	[14]	62	3 (4.8)
	2012–2016	Skåne	1.26–1.32	[11]	397	44 (11.1)
	2012–2016	Stockholm	Karolinska^h^	[11]	268	24 (9.0)
HACEK	2012–2017	Skåne and Stockholm	3.2	[20]	118	27 (22.9)

Abbreviations: HACEK, *Haemophilus*, *Aggregatibacter*, *Cardiobacterium*, *Eikenella*, *Kingella*; IE, infective endocarditis; NBHS, non–β-hemolytic streptococci.

^a^NBHS comprising the mitis, sanguinis, mutans, salivarius, and anginosus groups; for species see reference 10. Isolates of the bovis group were excluded.

^b^Only half of 2013.

^c^The end of the study period was 31 March 2016.

^d^All NBHS, including the bovis group.

^e^ *Streptococcus bovis–Streptococcus equinus* complex: *Streptococcus gallolyticus* subsp *pasteurianus*, *S. gallolyticus* subsp *gallolyticus, Streptococcus lutetiensis,* and *Streptococcus infantarius*.

^f^Including *Abiotrophia* (n = 19)*, Aerococcus* (n = 338)*, Gemella* (n = 87), and *Granulicatella* (n = 124).

^g^In this study all pathogens were regarded as “typical IE pathogens” [18].

^h^A tertiary center, not population based.

Changes introduced in the Duke-ISCVID criteria that were available for reanalysis in our cohorts were (1) the upgrading of *Streptococcus dysgalactiae, Staphylococcus lugdunensis,* and *Gemella* to “typical IE pathogens”; (2) the withdrawal of the demand for nonnosocomial acquisition and lack of a focal infection for *E. faecalis* to be regarded as a typical IE pathogen; (3) the addition of a positive PET-CT as a major imaging criterion; and (4) the addition of a CIED as a new predisposition minor criterion. The Duke-ISCVID criteria specify *Abiotrophia* and *Granulicatella* as typical IE pathogens, but our interpretation is that these organisms were implicitly included in the viridans streptococci in the previous criteria [2, 3], whereas *Gemella* was added to the list in the Duke-ISCVID criteria. There were no missing data for the variables regarded for reanalysis.

The clinical decision to treat a patient for IE was compared with classifications by the 2 criteria. The information on treatment as IE was lacking for one of the cohorts and this cohort was excluded from this analysis. The concordance between the clinical decision to treat as IE and the classification of patients as definite or not definite IE according to the 2000 modified Duke criteria and the Duke-ISCVID criteria was calculated and presented.

Values are given as numbers and shares of the total. Significance was defined as a *P* value <.05. GraphPad Prism software (version 9; GraphPad Software) was used for statistical calculations. All of the studies had been approved by relevant regional or national ethics boards, and no additional personal information was collected for the present study.

## RESULTS

### New Major and Minor Criteria

A total of 4050 episodes of bacteremia were reanalyzed (Table 2). Episodes caused by *S. lugdunensis, S. dysgalactiae, Gemella,* and *E. faecalis* (nosocomial acquired or with known focus) were eligible for reclassification from the minor to the major microbiology criterion since these species were changed to “typical IE pathogens” in the Duke-ISCVID criteria. Thus, those with 2 positive blood culture sets were transferred to the major microbiology criterion. Such reclassification occurred in 518 episodes (13% of all episodes). The majority of these reclassifications occurred in the *S. dysgalactiae* and *E. faecalis* cohorts (Table 2).

**Table 2.** Episodes of Bacteremia and Reclassification of Minor and Major Criteria

Microbiological Agent	Episodes of Bacteremia, No.	Change of Microbiology Minor to Major Criteria	PET-CT Providing New Major Criterion	CIED as New Minor Criterion
*Staphylococcus aureus*	1365	0	0	98
*Staphylococcus lugdunensis*	65	27	0	2
NBHS^a^	920	0	1	23
*Streptococcus*-like bacteria	568	22^b^	0	25
*Streptococcus dysgalactiae*	287	192	0	11
*Enterococcus faecalis*	727	277	0	49
HACEK	118	0	0	6

Abbreviations: CIED, cardiovascular implanted electronic device; CT, computed tomography; HACEK, *Haemophilus*, *Aggregatibacter*, *Cardiobacterium*, *Eikenella*, *Kingella*; NBHS, non–β-hemolytic streptococci; PET, positron emission tomography.

^a^NBHS comprising the mitis, sanguinis, mutans, salivarius, anginosus, and bovis groups; for species see references 10 and 17.

^b^ *Gemella* was changed to a typical pathogen in the 2023 Duke-International Society of Cardiovascular Infectious Diseases (ISCVID) criteria, *Abiotrophia* and *Granilucatella* were counted as typical pathogens in both 2000 modified Duke and 2023 Duke-International Society of Cardiovascular Infectious Diseases (ISCVID) criteria, and *Aerococcus* was regarded as nontypical in both criteria.

The performance of PET-CT without findings of IE was not systemically recorded in all cohorts but PET-CT or CT of the heart provided a new major criterion in in only 1 episode. The patient had a CIED in 322 episodes (8.0% of all episodes). In some of these patients another predisposing factor was already present, and the CIED therefore provided a new minor criterion, according to the Duke-ISCVID criteria, in 214 episodes (5.3%) (Table 2).

### Reclassification

**Figure 1.**
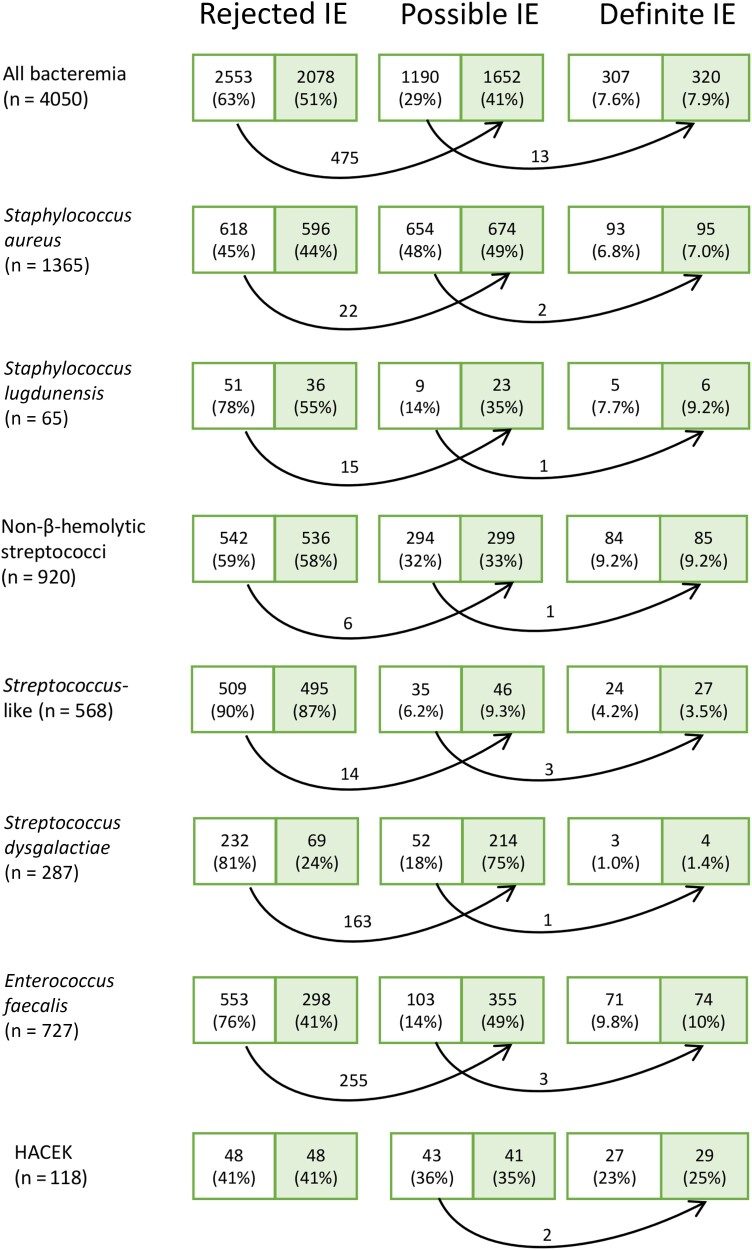
Displayed in the boxes are the numbers of episodes (and the share [percentage] of total episodes) assigned to rejected, possible, and definite infective endocarditis (IE). The left (*unshaded*) part of each box shows the result using the 2000 modified Duke criteria; the right (*shaded*) part, the result using 2023 Duke-International Society of Cardiovascular Infectious Diseases (ISCVID) criteria. Arrows indicate reclassification of episodes, with the number of reclassified episodes displayed above the arrow. Abbreviation: HACEK, *Haemophilus*, *Aggregatibacter*, *Cardiobacterium*, *Eikenella*, *Kingella.*

Applying the 2000 modified Duke criteria to all the cohorts resulted in 307 episodes (7.6%) of definite IE, 1188 (29%) of possible IE, and 2553 (63%) of rejected IE. With the Duke-ISCVID criteria, 13 episodes were reclassified from the possible category to definite IE, and 475 episodes from the rejected category to possible IE (Figure 1). The definite IE group thereby increased by 4.2%, and the possible IE group by 39%. Figure 1 shows the reclassification of episodes in the cohorts of patients with bacteremia caused by different bacteria. Table 3 shows a cross-tabulation of episodes with bacteremia classified as definite, possible, and rejected IE by the 2000 modified Duke criteria and the Duke-ISCVID criteria.

**Table 3.** Comparison of Infective Endocarditis Status by 2000 Modified Duke and 2023 Duke-International Society of Cardiovascular Infectious Diseases Criteria

Classification by Modified Duke Criteria	Bacteremia Episodes by Duke-ISCVID Criteria Classification, No.		
	Definite IE	Possible IE	Rejected IE
Definite IE	307	0	0
Possible IE	13	1190	0
Rejected IE	0	475	2078

Abbreviations: IE, infective endocarditis; ISCVID, International Society of Cardiovascular Infectious Diseases.

Features of the individual patients reclassified from possible to definite IE are given in Table 4. The proportion of cases reclassified from possible to definite IE was largest among patients with *Streptococcus-*like bacteremia, among whom 3 episodes (8.6% of possible IE episodes) were reclassified.

**Table 4.** Bacteremia Episodes Reclassified From Possible to Definite Infective Endocarditis in the 2023 Duke-International Society of Cardiovascular Infectious Diseases Criteria

Patient Age, y (Sex)	Bacteria	Treated as IE	Positive Cultures, No./Total No.	TEE or TTE Findings	PET-CT	Minor Criteria^a^	Acquisition	Surgery	Comments
74 (Male)	*Staphylococcus aureus*	Yes	4/4	TEE—Neg	ND	Fever, embolic event, ICD	Community	ICD extraction	No growth on extracted ICD
54 (Male)	*S. aureus*	No	4/4	TEE—Neg	ND	Fever, embolic event, ICD	Community	No	Antibiotic treatment for 12 d; no recurrence
85 (Female)	*Staphylococcus lugdunensis*	Yes	4/4	TEE—Veg	ND	Fever	Community	No	…
76 (Male)	*Streptococcus infantarius* sp (bovis group)	Yes	4/4	TEE—Neg	PVE	Fever, prosthetic valve	Community	No	Aortic root abscess; in-hospital death
88 (Male)	*Granulicatella adiacens*	Yes	1/4	TEE—Veg	ND	Microbiology, fever, PM	Nosocomial	No	No PM extraction
70 (Male)	*Gemella* sp	Yes	4/4	TEE—Veg	ND	Fever	Community	Yes	16S PCR on valve *Gemella* sp
77 (Female)	*Aerococcus urinae*	Yes	4/4	TEE—Veg	ND	Microbiology, fever, PM	Healthcare	No	No PM extraction
93 (Male)	*Streptococcus dysgalactiae*	Yes	4/4	TTE—Neg	ND	Fever, native valve disease, vascular phenomenon	Community	No	**…**
82 (Male)	*Enterococcus faecalis*	No	4/4	TEE—Neg	ND	Prosthetic valve, previous IE, fever, stroke	Nosocomial	No	…
83 (Female)	*E. faecalis*	Yes	4/6	TEE—Veg	ND	Predisposition, fever	Nosocomial	No	…
80 (Male)	*E. faecalis*	Yes	4/4	TTE—Neg	ND	Predisposition, fever, embolization	Nosocomial	No	…
73 (Male)	*Aggregatibacter* sp	Yes	1/4	TEE—Veg	ND	Fever, PM	Community	No	…
70 (Male)	*Aggregatibacter* sp	Yes	1/4	TEE—Veg	ND	Fever, PM	Community	PM extracted	16S PCR on PM *Aggregatibacter* sp

Abbreviations: CT, computed tomography; ICD, implantable cardioverter defibrillator; IE, infective endocarditis; ND, not done; Neg, negative; PCR, polymerase chain reaction; PET, positron emission tomography; PM, pacemaker; PVE, prosthetic valve endocarditis; TEE, transesophageal echocardiography; TTE, transthoracic echocardiography; Veg, vegetation.

^a^ICD and PM were new additions to the 2023 Duke-International Society of Cardiovascular Infectious Diseases (ISCVID) criteria.

### Agreement Between Diagnostic Criteria and Clinical Management

For all cohorts, except for the 2016 part of the *S. aureus* Skåne cohort [6], there was information about which episodes were diagnosed and treated as IE even though the 2000 modified Duke criteria for definite IE were not fulfilled. For this part of the study population (3508 episodes), 79 patients (2.2%) with episodes not fulfilling definite IE criteria were regarded as having IE based on being treated for IE (see above). Table 5 shows the consequences of the reclassification made by the Duke-ISCVID criteria in these patients. In Supplementary Table 1, the consequences of reclassifications are given for each bacterial species or group. If the clinical decision to treat the patient as having IE was used as the reference standard, the sensitivity of the 2000 modified Duke criteria was 77% compared with 80% for the Duke-ISCVID criteria, and this difference was not significant (*P* = .4 [Fisher exact test]).

**Table 5.** Concordance Between Diagnostic Criteria and Treatment for Infective Endocarditis

IE Classification by Criteria	Episodes, No. (%)	
	Treated as IE	Not Treated as IE
2000 Modified Duke criteria	Yes	No
Definite IE	267	0
Possible IE	79	841
Rejected IE	0	2321
2023 Duke-ISCVID criteria	Yes	No
Definite IE	278	1
Possible IE	68	1307
Rejected IE	0	1854

Abbreviations: IE, infective endocarditis; ISCVID, International Society of Cardiovascular Infectious Diseases.

The largest proportions of the episodes treated as IE despite not fulfilling Duke-ISCVID criteria for definite IE were found in *Streptococcus-*like and HACEK bacteremia, for which 41% and 29%, respectively, of patients treated as having IE did not receive a definite IE diagnosis according to criteria. Notably, 7 patients with *Aerococcus urinae* bacteremia had vegetations demonstrated by echocardiography and were treated as having IE but did not fulfil definite IE criteria in either 2000 modified Duke or Duke-ISCVID criteria. Of the 475 patients reclassified from rejected IE to possible IE, none received treatment for IE or were perceived as having IE.

The specificity of the Duke-ISCVID criteria for definite IE in relation to treatment for IE was marginally lower than that of the modified Duke criteria. Only 2 episodes were reclassified to definite IE despite not being treated for IE. One of these patients likely did not have IE since his infection was cured after only 12 days of antibiotic treatment and did not relapse (see Table 4). The specificity of the criteria went from 73% with the modified Duke criteria to 59% with Duke-ISCVID (*P* < .001 [χ^2^-test]) assuming that the possible and definite IE episodes were regarded as IE and the clinical decision to treat as IE was used as the reference standard.

## DISCUSSION

Applying the Duke-ISCVID 2023 criteria to our cohorts of patients who had bacteremia with IE-causing bacteria increased the proportion of patients assigned to the possible IE group by 39%, from 29% to 41%, of the entire cohort. This increase was mainly due to the recognition of *E. faecalis* and *S. dysgalactiae* as typical IE pathogens. When the Duke criteria were modified in 2000, a specific aim was to decrease the proportion of patients falling into the possible IE group [2]. With the inclusion of more bacteria as typical IE pathogens, without changing other criteria, the Duke-ISCVID criteria will classify many more patients as having possible IE. The typical patient with *S. dysgalactiae* bacteremia classified as possible IE in our study had cellulitis and fever. Such a patient has very low risk of having IE and would likely not benefit from workup (eg, with echocardiography) for possible IE. Thus, if more bacteria are included as typical IE pathogens, other changes are needed for the criteria not to include patients without IE in the possible IE group. For example, the usefulness of fever as a minor criterion in patients with bacteremia can be questioned, since fever and bacteremia are likely to display collinearity. This should be addressed in future studies, which need to include patients with suspected IE both with and without bacteremia. It is also problematic that IE can never be rejected in patients with bacteremia with a typical IE pathogen such as *S. dysgalactiae*, since the criteria for rejection demand that the bacteremia has to be caused by a “nontypical” IE pathogen [4]. Therefore, the Duke-ISCVID criteria will classify many patients with *S. dysgalactiae* and *E. faecalis* bacteremia as having possible IE, despite the extremely low probability for IE in a given patient.

Several studies have shown that the risk of IE is low in *E. faecalis* bacteremia of nosocomial origin and with known focus [11–13, 21, 22] as well as in *S. dysgalactiae* bacteremia [19, 23–25]. The study referred to in the Duke-ISCVID criteria to justify the inclusion of *S. dysgalactiae* as a typical IE pathogen is the only one reporting a much higher risk of IE in *S. dysgalactiae* bacteremia [4, 9]. It should be noted that this study was based on *International Classification of Diseases* codes and not on studies of medical records, thereby risking an increased proportion of misclassified episodes [9].

The Duke-ISCVID criteria assigned an additional 13 episodes (0.3% of all episodes) in our cohorts to the category of definite IE and increased the number of patients in this category from 307 to 320, an increase of 4.2%. Through the changes introduced in Duke-ISCVID criteria, 11 of 78 (14%) of patients who were treated for IE but did not fulfill 2000 modified Duke criteria for definite IE were reclassified to definite IE. Of course, it is not certain that the patients perceived as having IE really had the condition. Of those who were perceived as having IE, were treated for IE but not classified as definite IE by the Duke-ISCVID criteria, the majority had bacteremia with *S. aureus* (n = 18 [2.2% of all episodes]), *Streptococcus bovis* (n = 9 [4.3%]), HACEK (n = 11 [9.3%]), or *Streptococcus-*like bacteria (n = 19 [3.3%]). It was obvious that the performance of the Duke-ISCVID criteria could have been improved by the addition of *A. urinae* as a “typical pathogen.” By making this change to the criteria, 7 episodes with *A. urinae* bacteremia would have been reclassified from possible to definite IE. All of these 7 patients had imaging demonstrating IE and were treated as having IE.

The main strengths of the current study are that the cohorts are large and population based, and patient information was gathered through careful studies of medical records. In addition, the patients with bacteremia caused by these pathogens constitute a relevant study cohort for the ISCVID-Duke criteria since the suspicion of IE is often raised by the finding of a gram-positive or HACEK bacterium in a blood culture.

Our study also had many weaknesses; the most obvious one is that relevant IE investigations were not performed in all patients, thus risking the possibility that the IE diagnosis was missed in some episodes. The proportion of patients investigated with PET-CT was particularly low, reflecting the fact that this modality was not frequently used during the study periods. The recognition of PET-CT as a major criterion for IE was implemented in Sweden in 2021, after the study periods of the presented cohorts. Moreover, several new features of the Duke-ISCVID criteria could not be evaluated in the current study. As the most obvious, several important IE pathogens that changed status in the Duke-ISCVID criteria—such as coagulase-negative staphylococci and *Streptococcus agalactiae* [4]*—*were not included. Other changes in the Duke-ISCVID criteria (eg, definitions of glomerulonephritis) could not be evaluated owing to lack of data in the respective cohorts. Another weakness is that even if the cohorts were population based, more uncommon pathogens, such as HACEK and *Streptococcus-*like bacteria, were overrepresented relative to *S. aureus.* Therefore, the exact figures from this study cannot be extrapolated to the entire population, neither in Sweden nor in the rest of the world.

In conclusion, the changes introduced in the Duke-ISCVID criteria regarding microbiology and predisposition led to an increased proportion of patients with possible IE among those with gram-positive bacteremia. None of the episodes reclassified from rejected to possible IE was perceived as IE by the treating physician. The sensitivity was not significantly increased, and most patients perceived as having IE remained in the possible IE group. If other components of the criteria remain unchanged, we suggest that *S. dysgalactiae* should be removed from the list of typical pathogens, whereas *A. urinae* should be included [26]. The Duke-ISCVID criteria could likely be improved by repeated revisions based on validation studies. We believe, however, that larger changes to the diagnostic criteria for IE are needed to improve their performance. An increased emphasis could be put on the imaging criteria and the presence of an imaging major criterion together with bacteremia, irrespective of whether the bacteria are typical or nontypical IE pathogens, could constitute the basis for a definite IE diagnosis. Such a different set of criteria needs to be generated from large-volume, high-quality data, preferably from several countries and from many relevant healthcare settings.

## Supplementary Data

Supplementary materials are available at *Clinical Infectious Diseases* online. Consisting of data provided by the authors to benefit the reader, the posted materials are not copyedited and are the sole responsibility of the authors, so questions or comments should be addressed to the corresponding author.

## Supplementary Material

ciae040_Supplementary_Data

## Notes

***Acknowledgment.*** The authors acknowledge all coauthors of the studies that this reanalysis was based on.

***Financial support*.** This work was supported by the Region Halland Research Council (research funding to H. L.), the Foundation of Sparbanken Varberg (research funding to H. L.), the Swedish Government Funds for Clinical Research (ALF) (to M. R.), and Skåne University Hospital (research funds to M. R.).
