# Supplementary material for: Performance of the 2023 Duke-International Society of Cardiovascular Infectious Diseases Diagnostic Criteria for Infective Endocarditis in Relation to the Modified Duke Criteria and to Clinical Management—Reanalysis of Retrospective Bacteremia Cohorts
Source: Clin Infect Dis. 2024 Feb 8;78(4):956–63. doi: 10.1093/cid/ciae040 (PMC11006102; doi:10.1093/cid/ciae040)
Supplement: ciae040_Supplementary_Data [file ciae040_supplementary_data.docx]

| **Total** | Treated as IE | |  | ***S. aureus*** | Treated as IE | |  | ***S. lugdunensis*** | Treated as IE | |  | **NBHS** | Treated as IE | |
| --- | --- | --- | --- | --- | --- | --- | --- | --- | --- | --- | --- | --- | --- | --- |
| Modified Duke | Yes | No |  | Modified Duke | Yes | No |  | Modified Duke | Yes | No |  | Modified Duke | Yes | No |
| Definite IE | 267 | 0 |  | Definite IE | 53 | 0 |  | Definite IE | 5 | 0 |  | Definite IE | 84 | 0 |
| Possible IE | 79 | 841 |  | Possible IE | 18 | 366 |  | Possible IE | 1 | 8 |  | Possible IE | 21 | 273 |
| Rejected IE | 0 | 2321 |  | Rejected IE | 0 | 386 |  | Rejected IE | 0 | 51 |  | Rejected IE | 0 | 542 |
|  |  |  |  |  |  |  |  |  |  |  |  |  |  |  |
| Duke-ISCVID | Yes | No |  | Duke-ISCVID | Yes | No |  | Duke-ISCVID | Yes | No |  | Duke-ISCVID | Yes | No |
| Definite IE | 278 | 1 |  | Definite IE | 54 | 0 |  | Definite IE | 6 | 0 |  | Definite IE | 85 | 0 |
| Possible IE | 68 | 1307 |  | Possible IE | 17 | 380 |  | Possible IE | 0 | 23 |  | Possible IE | 20 | 279 |
| Rejected IE | 0 | 1854 |  | Rejected IE | 0 | 372 |  | Rejected IE | 0 | 36 |  | Rejected IE | 0 | 536 |
|  |  |  |  |  |  |  |  |  |  |  |  |  |  |  |
| ***Strep*-like** | Treated as IE | |  | ***S. dysgalactiae*** | Treated as IE | |  | ***E. faecalis*** | Treated as IE | |  | **HACEK** | Treated as IE | |
| Modified Duke | Yes | No |  | Modified Duke | Yes | No |  | Modified Duke | Yes | No |  | Modified Duke | Yes | No |
| Definite IE | 24 | 0 |  | Definite IE | 3 | 0 |  | Definite IE | 71 | 0 |  | Definite IE | 27 | 0 |
| Possible IE | 22 | 13 |  | Possible IE | 1 | 51 |  | Possible IE | 5 | 98 |  | Possible IE | 11 | 32 |
| Rejected IE | 0 | 509 |  | Rejected IE | 0 | 232 |  | Rejected IE | 0 | 553 |  | Rejected IE | 0 | 48 |
|  |  |  |  |  |  |  |  |  |  |  |  |  |  |  |
| Duke-ISCVID | Yes | No |  | Duke-ISCVID | Yes | No |  | Duke-ISCVID | Yes | No |  | Duke-ISCVID | Yes | No |
| Definite IE | 27 | 0 |  | Definite IE | 4 | 0 |  | Definite IE | 73 | 1 |  | Definite IE | 29 | 0 |
| Possible IE | 19 | 27 |  | Possible IE | 0 | 213 |  | Possible IE | 2 | 352 |  | Possible IE | 9 | 32 |
| Rejected IE | 0 | 495 |  | Rejected IE | 0 | 70 |  | Rejected IE | 0 | 298 |  | Rejected IE | 0 | 48 |

**Supplementary table 1.** Concordance between criteria for IE diagnosis and treatment for IE according to group or species of bacteria
